# Mulberry Leaf Extracts and Neochlorogenic Acid Ameliorated Scopolamine-induced Cognitive Dysfunction through Promotion of Adult Hippocampal Neurogenesis

**DOI:** 10.7150/ijms.136787

**Published:** 2026-08-24

**Authors:** Li-Sung Hsu, Yi-Ling Cheng, Yun-Ching Chang, Yi-Ju Lee, Chau-Jong Wang

**Affiliations:** 1 Institute of Medicine, Chung Shan Medical University, Taichung 402, Taiwan.; 2 Department of Medical Research, Chung Shan Medical University Hospital, Taichung, 402, Taiwan.; 3 Department of Health Industry Technology Management, Chung Shan Medical University, Taichung 402, Taiwan.; 4 Department of Pathology, Chung Shan Medical University Hospital, Taichung 402, Taiwan.; 5 Department of Pathology, Chung Shan Medical University, Taichung 402, Taiwan.

**Keywords:** mulberry leaf extracts (MLWE), neochlorogenic acid, scopolamine, adult hippocampus neurogenesis, brain-derived neurotrophic factor

## Abstract

Loss of neurogenesis leads to cognitive impairment and is one of the main causes of neuron degeneration diseases. Mulberry, an economic plant used as Chinese traditional herb, exhibits multiple biological properties. This study investigated the protective molecular mechanisms of mulberry leaf extracts (MLWE) and neochlorogenic acid (nCGA) on scopolamine-induced neuron cell death and cognitive dysfunction. Administration of MLWE and nCGA significantly improved cognitive functions in the Y-maze test of scopolamine-treated mice. MLWE and nCGA reversed scopolamine-reduced brain-derived neurotrophic factor (BDNF) expression and its downstream pathways. Immunohistochemical staining or Western blot analysis showed that MLWE and nCGA increased the expression of neurogenetic proteins such as nestin, NeuN, and doublecortin (DCX) in scopolamine-treated mice. They also reversed the hippocampus structure and neuronal proliferation, decreased Bax expression, and increased Bcl2 expression, thereby attenuating scopolamine-induced neural apoptosis. MLWE (0.5 mg m/L) and nCGA (10 μM) rescued scopolamine-induced cell death in SH-SY5Y cells. MLWE and nCGA repressed scopolamine-induced apoptosis, as evidenced by decreasing sub-G1 phase population through regulation of Bcl2 and Bax expression. Moreover, MLWE and nCGA promoted neurogenesis marker expression in SH-SY5Y cells. Our findings revealed that MLWE and nCGA significantly reversed scopolamine-induced neuron damage through enhanced adult hippocampus neurogenesis.

## Introduction

Neurodegeneration, which means loss of neurons especially in the central nerve system (CNS), leads to dementia, Alzheimer's disease, and Parkinson disease, is a major health issue worldwide 1. Neurons are generated in the subventricular zone of the lateral ventricles and the subgranular zone of the dentate gyrus in the hippocampus, and this process is called adult hippocampus neurogenesis (AHN) 2. Evidence indicated that enhanced AHN may prevent or rescue neurodegeneration diseases 2.

Flavonoids derived from vegetables, fruits, and traditional herbs exhibit neuroprotective effects through their anti-oxidant and anti-inflammation properties 3. Dietary flavonoids can ameliorate neurodegeneration diseases by stimulating the proliferation of neuronal stem cells in adult brain. Lee et al. demonstrated that hesperidin, a flavonoid found in citrus, enhanced hippocampus neurogenesis by activating brain-derived neurotrophic factor (BDNF)/ cAMP-response element-binding protein (CREB) pathway and increased cognitive functions in a mouse model of Alzheimer's disease 4. Cyperus rotundus extracts significantly promoted spatial memory and neuronal differentiation in mice injected with β-amyloid 5. Oral intake of icarisid II elevated b-catenin activity, promoted hippocampus neurogenesis, and subsequently recovered cognitive impairment in amyloid precursor protein (APP)/presenilin-1 transgenic mice 6. Karimipor et al. demonstrated that quercetin upregulated BDNF, nerve growth factor (NGF) and CERB expression, enhanced neurogenesis in dentate gyrus region in the hippocampus, and rescued learning and memory performance in mice with amyloid protein-induced Alzheimer's disease 7. Nuciferine leaf polyphenol extracts not only promoted hippocampus neurogenesis but also recovered scopolamine-induced cognitive disfunction 8, 9.

Mulberry, an economic plant widely distributed in tropical countries, is used as antibacterial, anti-rheumatic, anti-inflammation, and anti-lipidemia agents. Mulberry is used as traditional medicine, and all its parts, including fruits, leaves, bark, and roots, can treat different symptoms, such as perspiration and hypertension, and be used as throat rinse and analgesics 10, 11. Mulberry leaf extracts (MLWE) contain bioactive flavonoids that exert anti-inflammation, anti-obesity and anti-cancer properties as well as neuroprotective capacity 10, 11. Methanol extracts of mulberry leaves diminished amyloid beta-peptide-induced fiber formation and recovered cell viability in the presence of amyloid beta-peptide in cultured hippocampal neurons 12. By using *in silico* and *in vitro* assay, El-Hawary et al. showed that bioactive photochemicals, such as chrysin, resveratrol, and ferulic acid extracted from mulberry prevented the aggregation of beta-amyloids 13. Mulberry fruit and leaf extracts recovered neural activities and presynaptic and postsynaptic activities in obese mice 14 and rescued hydrogen peroxide-induced oxidative stress and apoptosis in cultured SH-SY5Y cells 15. Overall, mulberry extracts obviously elevated nerve growth factor (NGF), activated extracellular signal-regulated kinases, promoted neuron differentiation and proliferation in hippocampus region, and finally improved memory and learning abilities 16.

Previous reports have been demonstrated that scopolamine is widely utilized as a pharmacological tool to study neurogeneration disorders such as Alzheimer diseases 17. Scopolamine-induced oxidative stress, neuroinflammation, cholinergic dysfunction, promoted apoptosis of hippocampus neurons and eventually caused cognitive impairment and neurodegeneration 17. In this study, we investigated the protective effects of MLWE and neochlorogenic acid (nCGA) on scopolamine-induced cognitive impairment and explored their underlying mechanisms. Our results show MLWE and nCGA triggered neurogenesis in hippocampus region, enhanced BDNF pathways, reduced apoptosis, and improved cognitive impairment in scopolamine treated mice. Similarly, MLLE and nCGA protected scopolamine-induced cell apoptosis and enhanced neurogenesis markers expression in cultured SH-SY5Y cells. These findings suggest that MLWE and nCGA can promote hippocampus neurogenesis and prevent or treat neurodegeneration disorders.

## Materials and Methods

### Materials

Neochlorogenic acid (nCGA) (purity>99.0%) was purchased from Chengdu Alfa Biotechnology (Chengdu, China). Scopolamine and tacrine were obtained from Sigma-Aldrich (St. Louis, MO, USA). Anti-nestin and anti-Doublecortin (DCX) antibodies were purchased from Abcam (Cambridge, UK). Phospho-TrkB and TrKB antibodies were obtained from ABclonal (Massachusetts, USA). Phospho-AKT, AKT and Phospho-CREB antibodies were purchased from Cell Signaling Technology (Boston, MA, USA). Anti-Bcl2 and anti-Bax were obtained from Santa Cruz Biotechnology (Santa Cruz, CA, USA).

### Preparation of mulberry leaf water extract (MLWE)

Mulberry (*Morus alba* L.) leaves are obtained from contracted mulberry farms (Dadu Township, central Taiwan) in June every year. About 100 g of dry mulberry leaves were added into 3000 mL of water and boiled for 1 h. After cooling, the solution was filtered to remove debris and then lyophilized using a vacuum freeze-drying system. The dried powders were stored at -80 °C. During each experiment, the powders were dissolved in water to the indicated working concentration. The bioactive compounds were determined by high-performance liquid chromatography (HPLC) combined with liquid chromatography-mass spectrometry (LC-MS) analyses. Briefly, polyphenols of MLWE were measured by Hitachi HPLC system (D-7000, Hitachi, Danbury, CT, USA) equipped with an ultraviolet detector (L-4250) and a Mightysil RP-18 GP 250 column (Kanto, Tokyo, Japan) system. The mobile phase contained two solvents: (A) acetic acid/water (2 : 98, v/v) and B (0.5% acetic acid in water/acetonitrile (50 : 50, v/v). The gradient program for elution was set as: 100% solvent A (0 min), 70% solvent A and 30% solvent B (5 min), 65% solvent A and 35%solvent B (50 min), 60% solvent A and 40% solvent B (55 min), and 100% solvent B (60 min). The elution rate was 1 mL/min and polyphenols were measured at 280 mM. The total polyphenols of each preparation was detected by Folin-Ciocalteu technique as previous described 18, 19. The polyphenols were further identified by LC-MS method 18, 19. The major polyphenols identified of MLWE were: (1) 0.355% neochlorogenec acid (nCGA), (2) 0.317% cryptochlorogenic acid, (3) 0.238% chlorogenec acid, (4) 0.092% rutin, (5) 0.056% isoquercitrin, (6) 0.053 astragalin 19.

### Animal model

Forty 6-weeks old male C57BL/6 mice were divided into five groups (each group contains 8 mice): (1) control, (2) scopolamine group: weekly intraperitoneal injection of 100 μL of scopolamine (3 mg/kg) for 6 weeks, (3) tacrine group: daily intraperitoneal injection of 100 μL of tacrine (10 mg/kg) plus weekly intraperitoneal injection of 100 μL of scopolamine (3 mg/kg) for 6 weeks, (4) MLWE group: daily oral administration of 0.5% MLWE plus weekly intraperitoneal injection of 100 μL of scopolamine (3 mg/kg) for 6 weeks, and (5) nCGA group: daily oral administration of 0.1 g/kg nCGA plus weekly intraperitoneal injection of 100 μL of scopolamine (3 mg/kg) for 6 weeks. These dosage for scopolamine and tacrine was selected according to previous report 9. The administration concentration of MLWE and nCGA was according to previous report 20 All procedures were approved by the institutional animal care and use committee of Chung Shan Medical University. The IACUC No. was 2812.

### Y maze analysis

Y maze analysis was conducted as previously described 9. Behavioral experiments were conducted at week 0, 3, and 6. For spontaneous alternation, the mice were placed in the center of Y-maze apparatus and allowed to explore the maze for 10 min. For novel arm tests, one arm was closed, and the mice were allowed to acclimate for 3 min. For each experiment, the closed arm was opened and allowed the mice to explore for 7 min. Spontaneous alternation and novel arm test were conducted as previously reported 9.

### Terminal deoxynucleotidyl transferase dUTP nick-end labeling (TUNEL) assay

About 5 μm paraffin-embedded brain slides derived from mice with indicated treatment were deparaffined and rehydrated. Antigen was retrieved by boiling in 10 mM citrate buffer (pH 6.0) at 100 °C for 20 min and added with 100 mL of Proteinase K solution at 37 °C for 30 min. The slides were washed with phosphate buffered saline (PBS) containing 0.1% Tween-20 (PBST), added with 100 μL of TdT equilibration buffer, and incubated at 37 °C for 30 min. The slides were then added with labeling working solution, incubated again at 37 °C for 90 min, and finally stained with DAPI solution. Images were captured using TISSUE GNOSTICS TissuFAXS cytometry and analyzed by TissueFAXS software.

### Immunohistochemical (IHC) assay

Paraffin-embedded 5 μM brain section was deparaffinized, rehydrated by sequentially incubating with ethanol, and treated with 3% H_2_O_2_ for 10 min to suppress endogenous peroxidase activity. Antigen was retrieved by boiling at 100 °C for 20 min in 10 mM citrate buffer (pH 6.0). The sections were washed with phosphate-buffered saline (PBS) and incubated with antibodies against DCX, nestin, or neuro N at 4 °C overnight. After washing with PBS, the sections were reacted with horseradish peroxidase-conjugated secondary antibodies. Positive signals were developed by UltraView Universal DAB Detection kit, and counterstaining with hematoxylin was conducted. Images were captured at 100 or 200 × magnification.

### Enzyme-link immunosorbent assay (ELISA)

The BDNF level was measured by mouse BDNF ELISA Kit (Arigo) according to manufacturer's protocols. Briefly, 50 μL serum derived from indicated treated mice was added into 96 well plate and then 50 μL antibody cocktail was added to each well. The plate was rotated at 400 rpm at room temperature for1 h. After washed with washing buffer for three times, 100 mL TMB Development Solution was added and incubated at room temperature in the dark for 10 min. The reaction was terminated by adding Stop solution. The BDNF level was calculated by absorbance of 450 mM.

### Bromodeoxyuridine (BrdU) incorporation assay

Mice were injected intraperitoneally (IP) with 75 mg/kg BrdU twice with 2 h interval. At 24 hours post-IP, the mice were sacrificed, and the brain sections were subjected to IHC analysis using anti-BrdU antibody. Positive signals were developed with UltraView Universal DAB Detection Kit.

### Cell culture

Human neuroblastoma SH-SY5Y cells were purchased from the American Type Culture Collection (ATCC) and maintained in Dulbecco's modified Eagle medium (DMEM) supplementary with 10% fetal bovine serum, 2 mM L-glutamine, 1% penicillin/streptomycin, and 1% non-essential amino acid. The cells were cultured at 37 °C with 5% CO2.

### Cytotoxicity assay

A total of 3 × 10^6^ cells of SHSY-5Y cells were seeded into 23-well plates and treated with MLWE (0, 0.5, 1, 2, 3, and 4 mg/mL), nCGA (0, 10, 50, 10, 150, and 200 μM), or scopolamine (0, 2, 4, 8, 16, and 20 mM) for 24 h. The treated concentration was selected as previous described 9, 33. For combined treatment, the cells were treated with 8 mM scopolamine alone or plus 0.5 mg/ML MLWE or 10 μM nCGA for 24 h. After treatment, the medium containing 10% 3-(4,5-dimethylthiazol-2-xl)-2,5 diphenyl-tetrazolium bromide (MTT) was added to each well and then incubated for additional 2 h. The purple residue was dissolved in isopropanol, and absorbance was recorded at 570 nm. Cell viability was measured by the following: OD570 of indicated treated group/OD570 of vehicle group × 100.

### Flow cytometry analysis

A total of 1 × 10^6^ SH-SY5Y cells were treated with vehicle, 8 mM scopolamine with or without 0.5 mg MLWE, or 10 μM nCGA for 24 h. The cells were stained with propidium iodide for 15 min in the dark. Cell cycle distribution was measured by BD Biosciences FACScan system and analyzed using CellQuestTM Pro software.

### Western blot analysis

The brain tissues and SH-SY5Y cells after indicated treatment were lysed using RIPA lysis buffer containing proteinase inhibitors. Protein concentration was detected by Bio-Rad protein assay kit. A total of 30 μg of protein was separated by polyacrylamide gel and transferred onto polyvinylidene difluoride membrane. The membrane was blocked by phosphate buffered saline (PBS) containing 0.1% Tween-20 (PBST) and 5% non-fat milk at room temperature for 1 h. After washing with PBST three times, the membrane was incubated with indicated first antibody at 4 °C for overnight. After washing with PBST, the membrane was added with PBST containing anti-rabbit or anti-mouse second antibodies conjugated with horseradish peroxidase and detected by enhanced chemiluminescent kit. Glyceraldehyde-3-phosphate dehydrogenase (GAPDH) or actin was used as loading control.

### Statistical analysis

All data were obtained from at least three independent experiments and represented by means and standard deviation. Data within groups were analyzed by one-way ANOVA with Duncan's multiple range test. Student's t-test was used to compare differences between two groups. All statistical analysis was using SPSS version 12 software. A p value < 0.05 indicated significant.

## Results

### MLWE and nCGA alleviated scopolamine-induced cognitive impairment

No overt alternation of serum biomarkers and food intake were found in control, scopolamine, tacrine, MLWE, and nCGA groups (Supplementary Figure 1A and B). On the other hand, body weight of tacrine group was significantly lower than control groups from weeks 3 onward. At week 6, the body weight of the MLWE group was significantly lower than those of the control group (Supplementary Figure 1C). A previous report demonstrated that scopolamine induced spatial recognition memory deterioration 17. Y-maze assay was conducted to determine whether MLWE and nCGA ameliorated scopolamine-induced cognitive impairment. The structure of nCGA was presented in Fig. 1A. As shown in Fig. 1B, scopolamine significantly decreased spontaneous alternation (short term memory) at weeks 3 and 6, and this phenomenon was recovered by treatment with MLWE and nCGA. In addition, duration of novel arm visit, distance traveled of novel arm, and percentage of novel arm entries at weeks 3 and 6 were measured. Treatment with MLWE and nCGA significantly recovered the scopolamine-induced phenomenon. Tacrine, a clinical anti-dementia drug, was used as positive control. At week 3, MLWE and nCGA was more effective than tacrine in behavior tests such as spontaneous alternation, duration of novel arm, and distance of novel arm (Fig. 1C-E).

### MLWE and nCGA attenuated scopolamine-induced apoptosis

The H and E staining results showed that scopolamine disrupted the territories of hippocampus regions, whereas MLWE, nCGA, and tacrine recovered scopolamine-induced phenomenon (Supplementary Fig. 2). Scopolamine also triggered apoptosis in hippocampus regions, as evidenced by TUNEL assay (Fig. 2A). Treatment with NLE and nCGA obviously reversed the scopolamine-induced phenomenon, and they also significantly suppressed pro-apoptosis marker Bax and increased anti-apoptosis marker Bcl2 expression compared with the scopolamine group (Fig. 2B).

### MLWE and nCGA enhanced brain-derived growth factor (BDNF) signals and promoted neurogenesis

BDNF signals play a critical role in neuron generation and proliferation 21. ELISA analysis was conducted to test whether MLWE and nCGA affect the BDNF level. As shown in Fig. 3A, scopolamine significantly reduced BDNF level, whereas MLWE or nCGA recovered the BDNF level. Western blot analysis also revealed that scopolamine downregulated BDNF expression and its downstream targets, whereas MLWE and nCGA significantly restored BDNF and activated TrkB, Akt, and CREB activities compared with the scopolamine group (Fig. 3B).

BrdU incorporation assay was performed to test whether MLWE and nCGA trigger neurogenesis. Scopolamine decreased BrdU positive signals compared with the control group. Treatment with MLWE or nCGA enhanced BrdU signals in the hippocampus regions (Fig. 4A). Immunohistochemical staining indicated that MLWE and nCGA elevated DCX and NeuN expression in the hippocampus regions (Fig. 4B and C). Western blot analysis also revealed that MLWE and nCGA recovered the expression of scopolamine-repressed neurogenesis markers such as nestin, DCX, and NeuN (Fig. 4D). Previous report indicated that administration of scopolamine for 6 weeks promoted β-amyloid expression 22. To test whether MLWE and nCGA affected neurodegeneration proteins expression, Western blot analysis was conducted. As shown in supplementary Fig. 3, no overt alternation of β-amyloid or phosphorylated tau protein expression in the presence of scopolamine alone or combined with tacrine, MLWE, or nCGA.

### MLWE and nCGA reversed scopolamine-induced apoptosis in cultured SH-SY5Y cells

Cultured SH-SY5Y cells were used to investigate the effects of MLWE and nCGA on scopolamine-induced phenomenon. SH-SY5Y cells were treated with different concentrations of MLWE, nCGA, or scopolamine for 24 h to test the cytotoxicity. Cell viability was assessed by MTT assay. As shown in Fig. 5, MLWE significantly reduced cell viability in proportion to concentration. nCGA at concentrations more than 100 μM and scopolamine at concentrations over 8 mM significantly inhibited cell proliferation. Next, we selected 0.5 mg/mL MLWE and 10 μM nCGA for further study.

To determine whether low dose MLWE or nCGA recovered scopolamine-induced cytotoxicity of SH-SY5Y, flow cytometry assay was conducted. The results of flow cytometry showed that 8 mM scopolamine combined with 0.5 mg/mL MLWE or 10 μM nCGA. MLWE or nCGA significantly recovered cell viability and apoptosis (sub-G1 phase population) compared with the scopolamine group (Fig. 6A). The Western blots also showed that MLWE or nCGA restored Bcl2 and repressed Bax expression compared with the scopolamine group (Fig. 6B).

### MLWE and nCGA ameliorated neurogenesis in scopolamine treated SH-SY5Y cells

Western blot analysis was conducted to explore whether MLWE and nCGA affect BDNF signals. Scopolamine obviously attenuated BDNF signals, whereas MLWE increased BDNF expression and activated TrkB and Akt compared with the scopolamine group. No overt alternation of nCGA on BDNF signals was found (Fig. 7). In addition, scopolamine downregulated neurogenesis markers such as nestin, DCX, and NeuN, but their expression was recovered by MLWE and nCGA (Fig. 8).

## Discussion

Cognitive impairment is one of the major health burdens worldwide. Fruits and vegetables, which have abundant flavonoids, have benefits for neurodegeneration disorders. Herein, we demonstrated that MLWE and nCGA significantly ameliorated scopolamine-induced cognitive impairment by preventing apoptosis, elevating BDNF signals, and promoting neurogenesis in the hippocampus regions *in vivo*. *In vitro* study also revealed that MLWE obviously reversed scopolamine-induced phenomenon.

Mulberry, an economic shrub mainly distributed in Asia, functions as traditional medicine to treat pain, parasite infection, and hypertension 10. Extracts from mulberry can prevent dementia by increasing memory ability. Administration of mulberry extract increased memory ability and prevented apoptosis by attenuating oxidative stress in the hippocampus region of mice with vascular dementia 23. Kim et al. showed that mulberry extracts recovered β-amyloid-induced cognitive impairment and oxidative stress through upregulation of glycogen synthase kinase 3β 14. Moreover, mulberry fruit extract attenuated β-amyloid-induced neuroinflammation and oxidative stress and then improved learning and memory abilities in animal models of Alzheimer's disease 24, 25. The present findings also 24, 25 abilities in the Y-maze test. Hence, MLWE and nCGA enhanced hippocampus cognitive function.

The hippocampus is the most important region for executive memory and learning. Loss of neurons in the hippocampus due to aging, oxidative stress, or inflammation leads to neurodegeneration diseases. Adult hippocampus neurogenesis (AHN), which means generation of new neurons in the hippocampus regions, alleviates brain disorder 26. Promotion of AHN provides a new strategy for prevention or treatment of cognitive impairment. Hesperidin recovered valproic acid or methotrexate-induced cognitive impairment through AHN 27, 28. Lee et al. demonstrated that oral administration of nuciferine leaf polyphenol extract (NLPE) significantly increased the number of neurons in the subgranular zone 8. In the present work, MLWE and nCGA promoted hippocampus neurogenesis as evidenced by enhanced BrdU incorporation. Moreover, MLWE and nCGA enhanced the expression of differentiated neuron markers, such as DCX, nestrin, and Neu N, *in vivo* and *in vitro*. The TUNEL staining and Western blot analysis results showed that MLWE and nCGA also prevented apoptosis in response to scopolamine. Overall, our findings indicated that MLWE and nCGA alleviated scopolamine-induced cognitive impairment by promoting AHN and reducing apoptosis.

BDNF plays a critical role in learning and memory in the hippocampus region 21. By binding to its receptor (tropomyosin-related kinase B receptor; TrkB), BDNF promoted the phosphorylation of CREB through the activation of AKT mitogen-activated protein kinase (MAPK) 21. The phosphorylated CREB bounds to and upregulated genes involved in cognitive functions 29, 30. Sim et al. reported that mulberry fruit extract (MFE) diminished glutamate-induced oxidative stress, promoted BDNF expression, and increased Trk, Akt, and CREB activities in hippocampal neuronal HT-22 cells 31. Treatment with MFE reversed scopolamine-induced memory loss *in vivo* 31. In alloxan-induced diabetic mice, MFE significantly ameliorated hyperglycemia-induced cognitive impairment, elevated acetylcholine level, and promoted the BDNF/CREB pathway 32. Flavonoids extracted from Cynomorium songaricum enhanced cognitive functions in mice with Alzheimer's disease by activating the BDNF/TrkB pathway 33. In the present study, while MLWE and nCGA significantly promoted BDNF signals *in vivo*, however, nCGA showed no obvious effect on BDNF signaling in SH-SY5Y cells *in vitro*. These observations suggested that the stimulation of hippocampal neurogenesis by nCGA may not be mediated through direct activation of the BDNF pathway in neurons. Instead, it might involve indirect systemic regulations, such as microenvironmental changes or alternative neurotrophic cascades, which warrant further investigation in future studies. Another possible explain was the concentration of nCGA utilized in our *in vitro* experiments was below the threshold required to trigger detectable BDNF signaling cascades in SH-SY5Y cells. Future studies utilizing a wider dose-response range or alternative cell models are needed to fully clarify this mechanism.

## Conclusion

In summary, our findings indicated that MLWE and nCGA effectively ameliorated scopolamine-induced neurotoxicity and cognitive impairment. They trigger AHN and prevent apoptosis in scopolamine-treated groups, as evidenced by increased BrdU positive signals, elevated neuronal differentiation marker expression, and modulated apoptosis-related protein expression. Our findings revealed that administration of MLWE or nCGA may reduce cognitive impairment by promoting AHN.

## Supplementary Material

Supplementary figures.

## Figures and Tables

**Figure 1:**
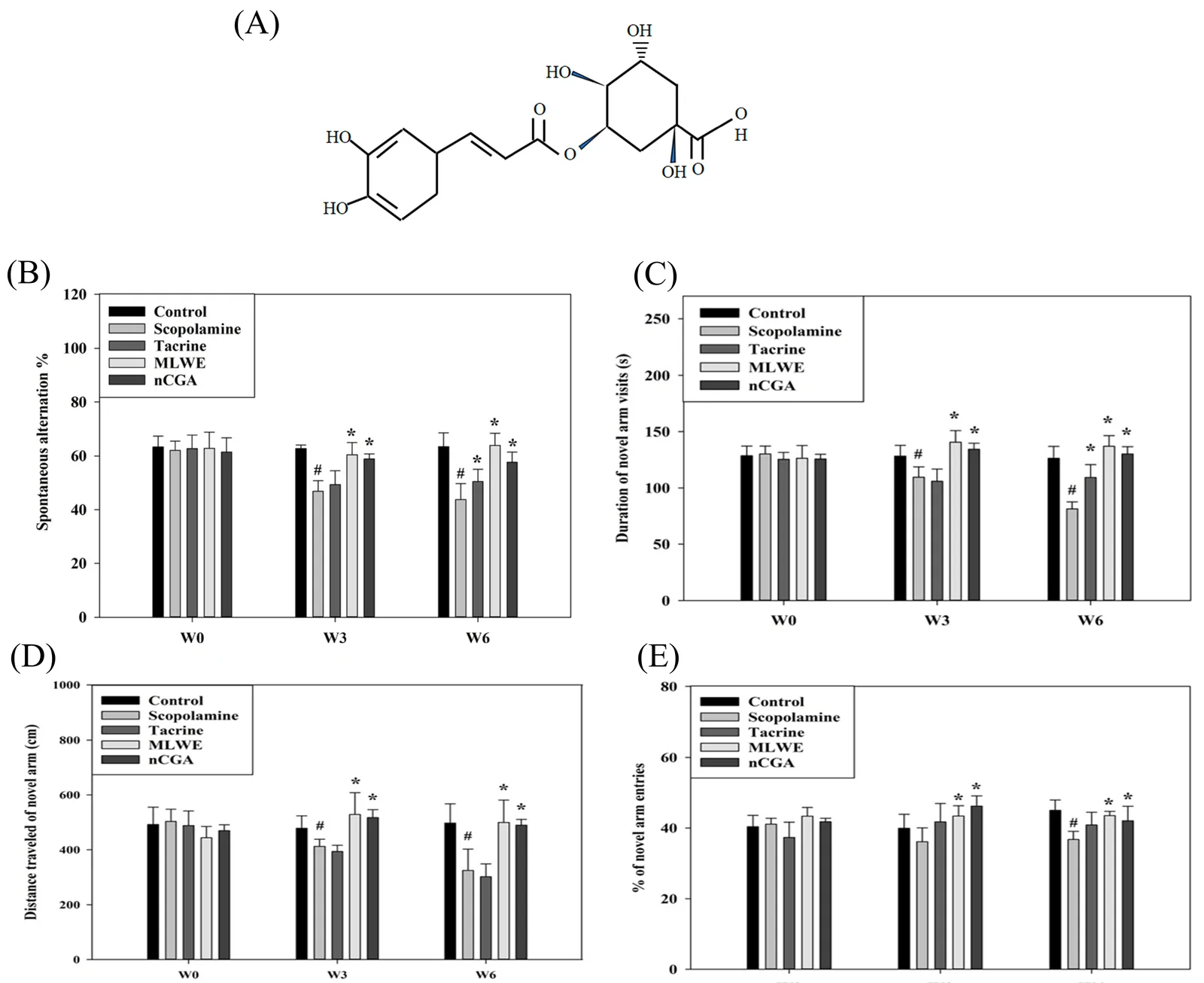
MLWE and nCGA recovered cognitive impairment in scopolamine-treated mice. Mice were subdivided into five groups as described in Materials and methods section. (A) The structure of nCGA. Cognitive functions such as (B) spontaneous alternation, (C) duration of novel arm, (D) distance of novel arm, and (E) % of novel entries were analyzed by Y-maze test. Data represented means ± standard deviation (SD). # p < 0.05, compared with the control group and * p < 0.05, compared with the Scopolamine group.

**Figure 2:**
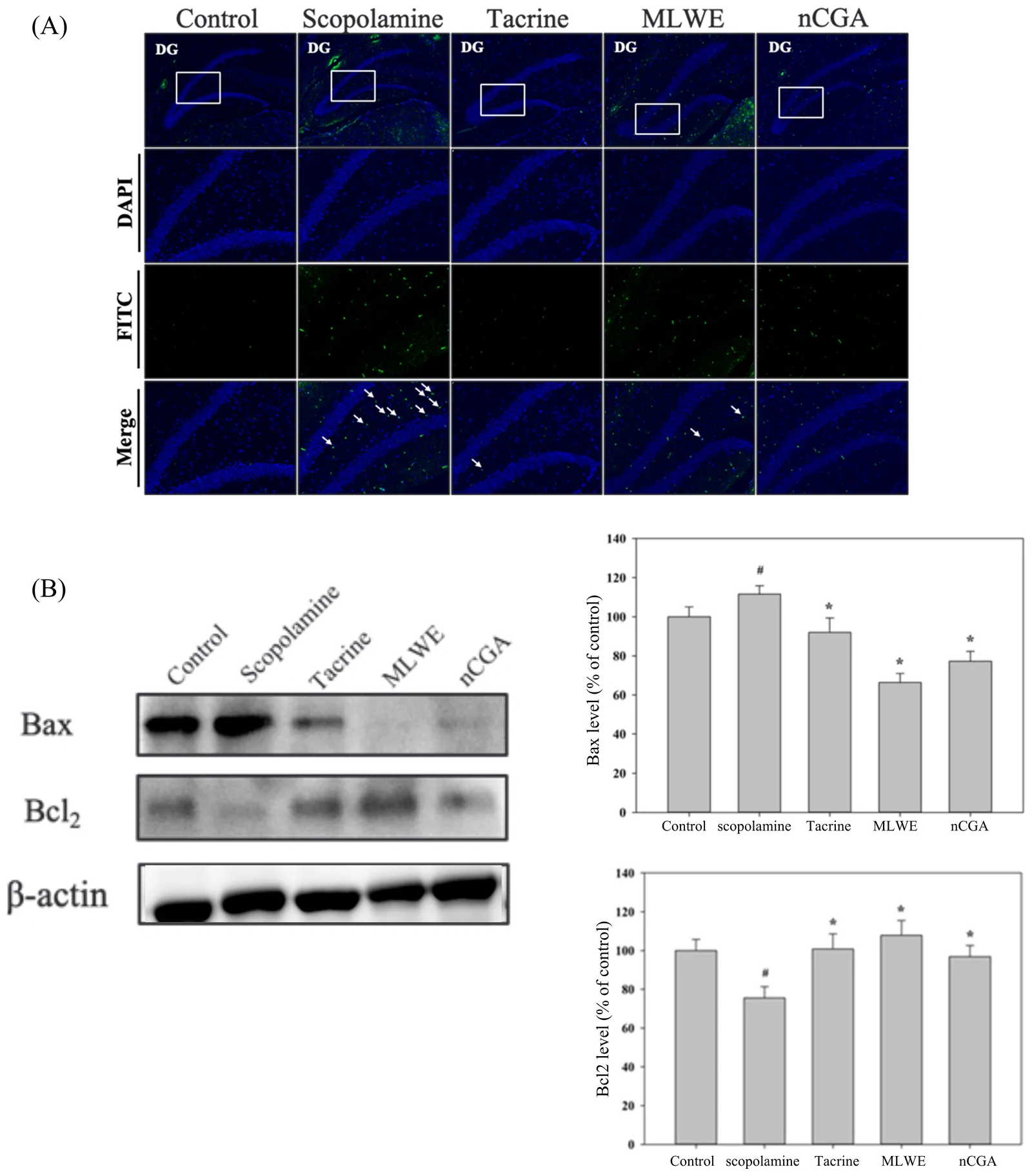
MLWE and nCGA reduced scopolamine-induced apoptosis in the hippocampus regions of mice. Mice were subdivided into six groups as described in Materials and methods section. (A) The brain sections were subjected into fluorescent TUNEL assay. Arrows indicated the positive signals for TUNEL assay. (B) A total of 30 μg brain proteins derived from indicated treatment were performed Western blot using Bcl2 or Bax antibodies. Data are shown as means ± SD. # p < 0.05, compared with the control group and * p < 0.05, compared with the scopolamine group.

**Figure 3:**
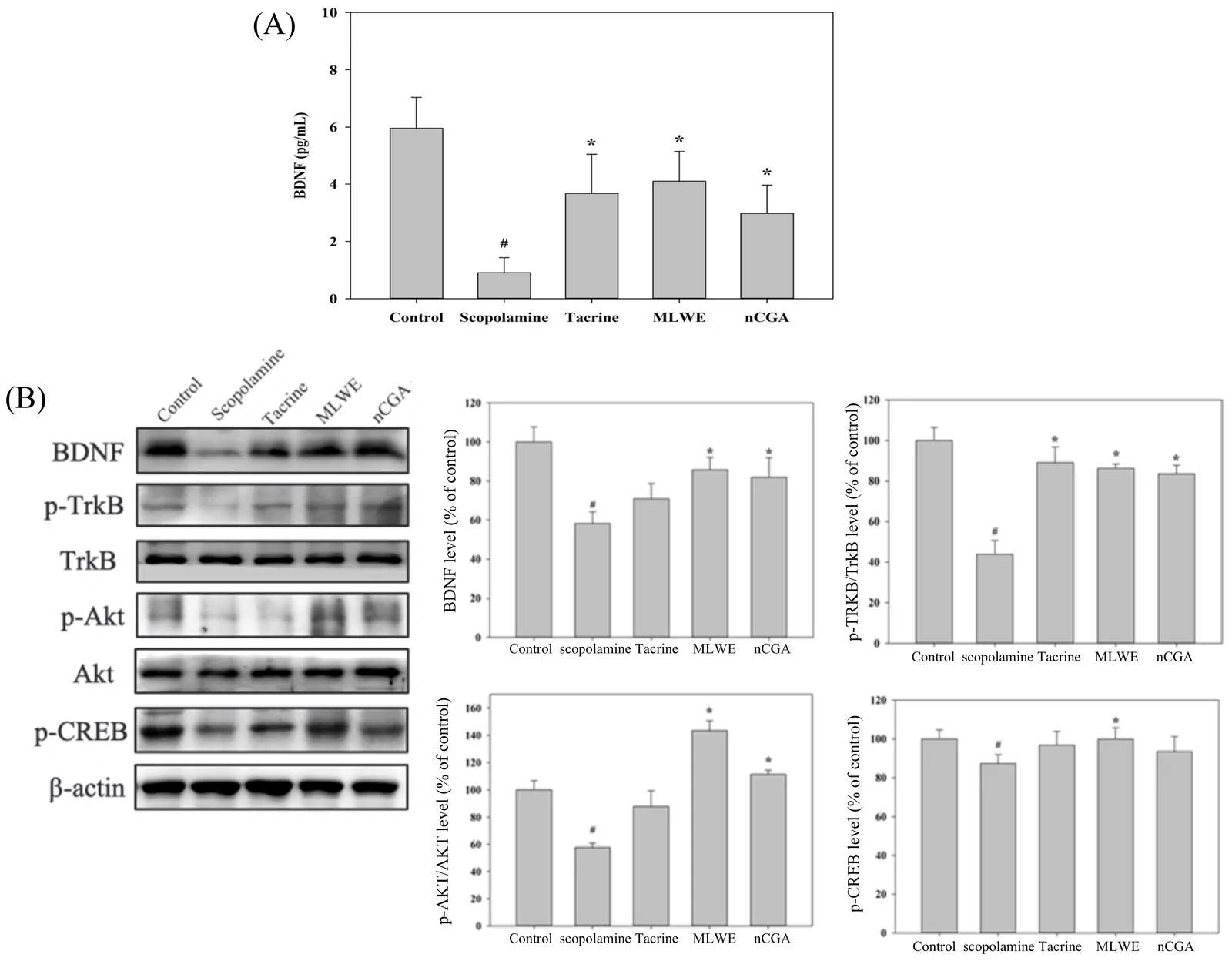
MLWE and nCGA elevated BDNF signals in scopolamine-treated mice. (A) Blood BDNF level from indicated treated groups were analyzed by ELISA assay. (B) BDNF downstream targets were measured by Western blot analysis. Data are shown as means ± SD. # p < 0.05, compared with the control group and * p < 0.05, compared with the Scopolamine group.

**Figure 4:**
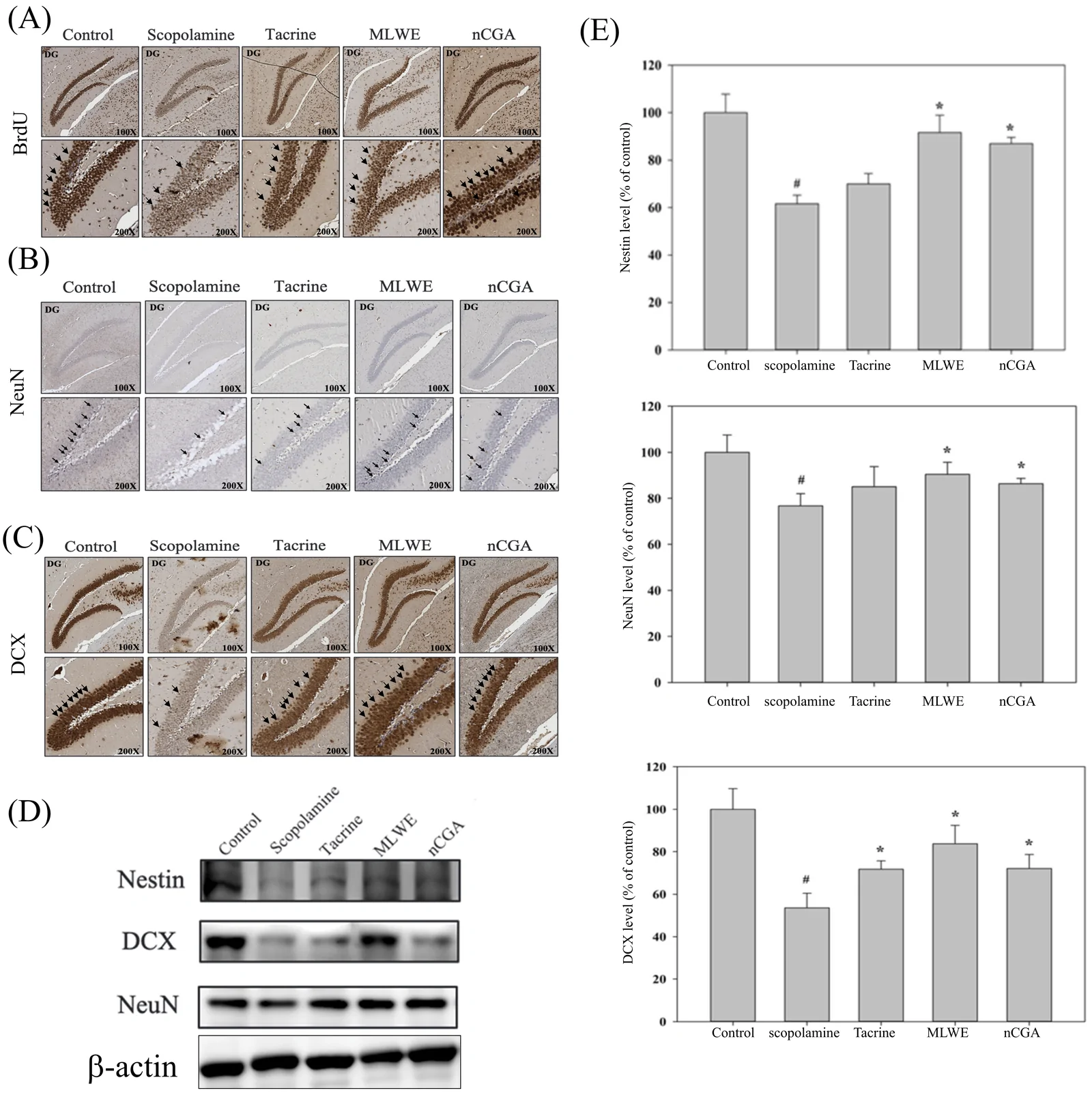
MLWE and nCGA promoted adult hippocampus neurogenesis (AHN) and neural differentiation. (A) Cell proliferation was detected by BrdU incorporation in brain sections. Immunohistochemical staining of (B) DCX and (C) Neu N of brain sections from indicated treated groups. Arrows indicated positive signals. (D) Hippocampus tissue extracts from indicated treated groups were subjected to western blotting to analyze nestin, DCX and NeuN expression. (E) Data are shown as means ± SD. # p < 0.05, compared with the control group and * p < 0.05, compared with the Scopolamine group.

**Figure 5:**
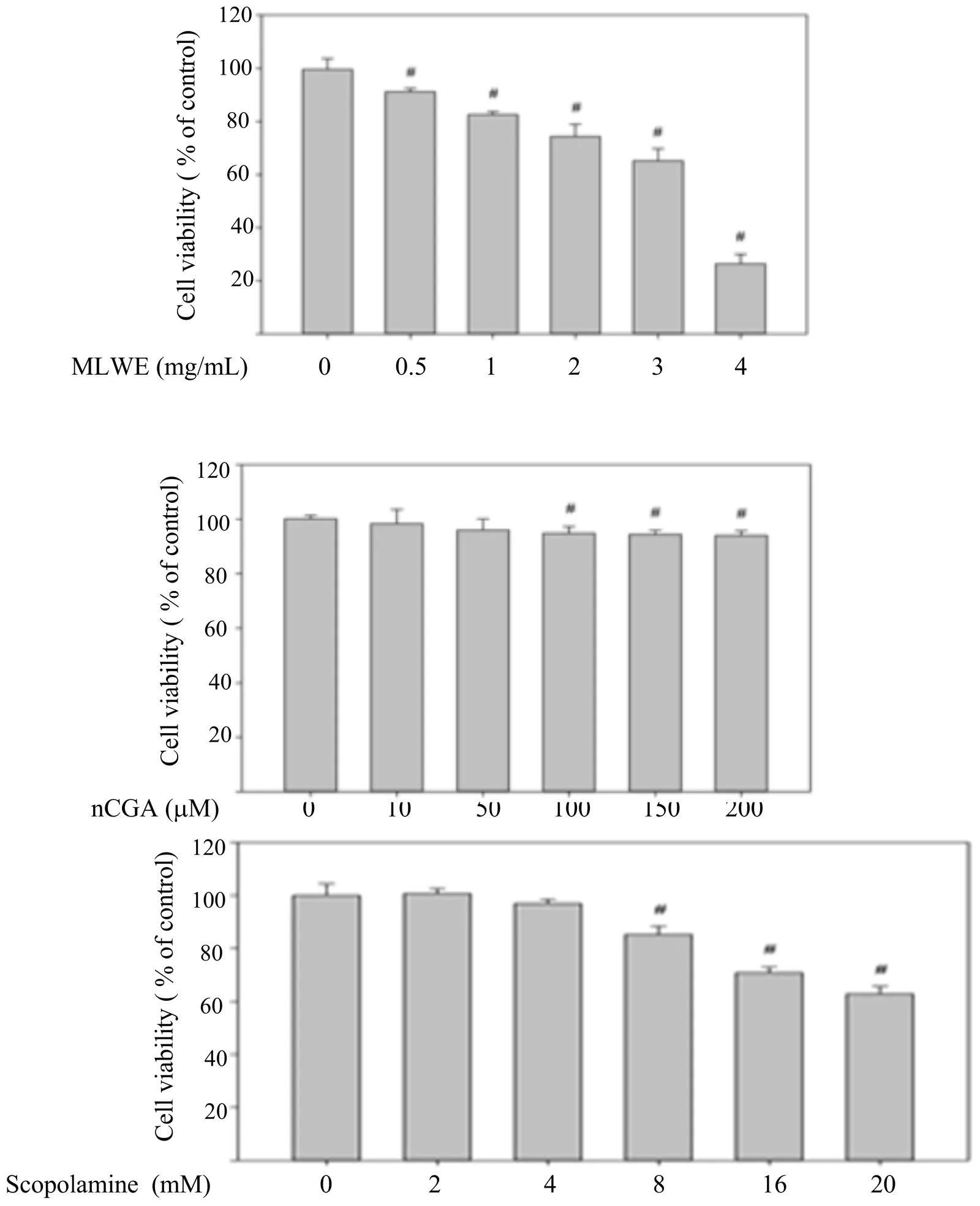
Cytotoxicity of scopolamine, MLWE, and nCGA of SH-SY5Y cells. Human neuroblastoma SH-SY5Y cells were treated with indicated concentration of MLWE, nCGA and scopolamine for 24 h, cell viability was detected by MTT assay. Data are shown as means ± SD. # p < 0.05, compared with the control group and * p < 0.05, compared with the Scopolamine group.

**Figure 6:**
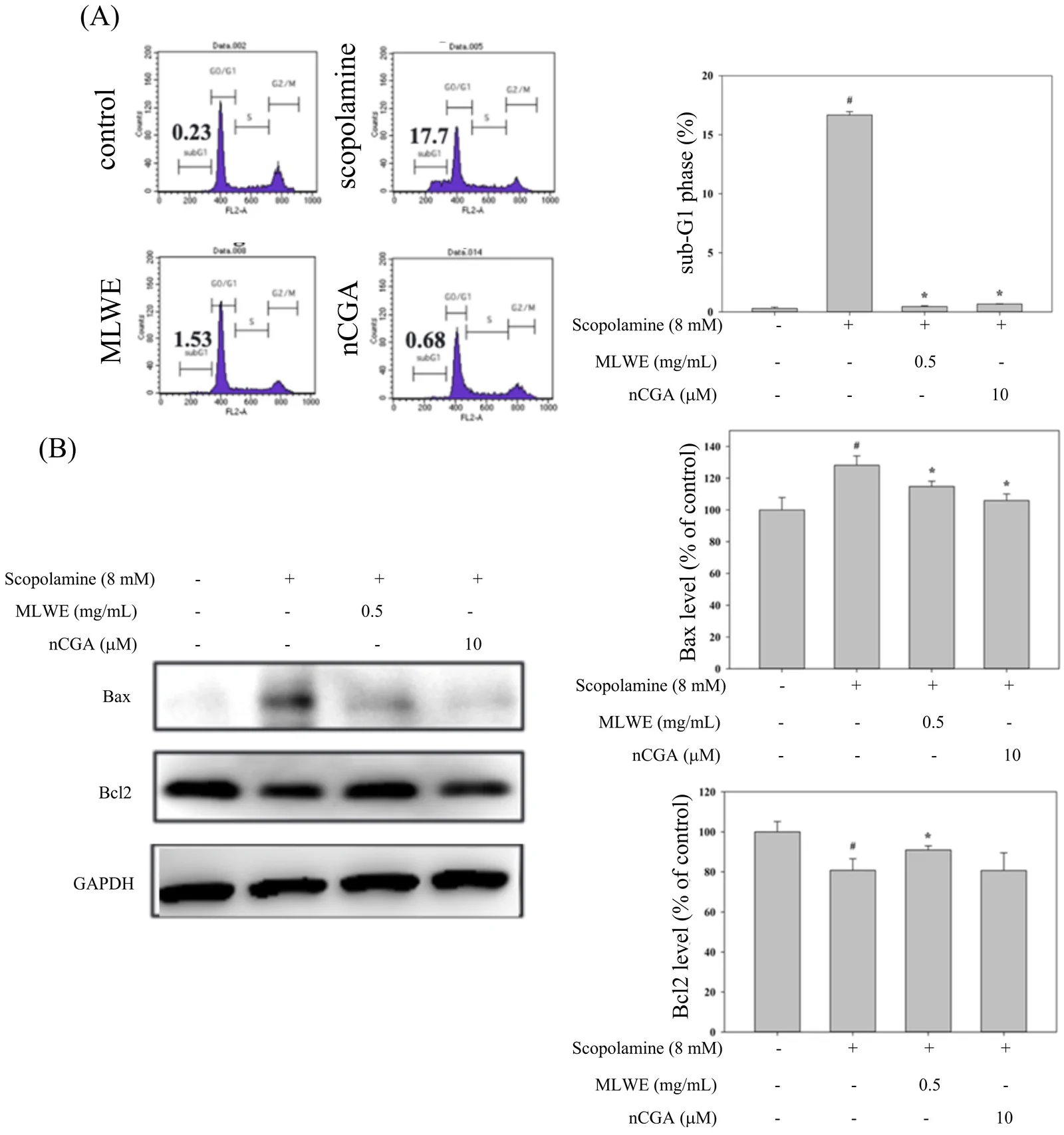
MLWE and nCGA recovered scopolamine-induced cell death of SH-SY5Y. SH-SY5Y cells treated with scopolamine (8 mM) alone or combined with MLWE (0.5 mg/mL) or nCGA (10 μM) for 24 h, the sub-G1 phase (apoptosis) was measured by propidium iodide staining (A). (B) Western blot analysis of Bcl2 and Bax was conducted in SH-SY5Y cells with indicated treatment. Data are shown as means ± SD. # p < 0.05, compared with the control group and * p < 0.05, compared with the Scopolamine group.

**Figure 7:**
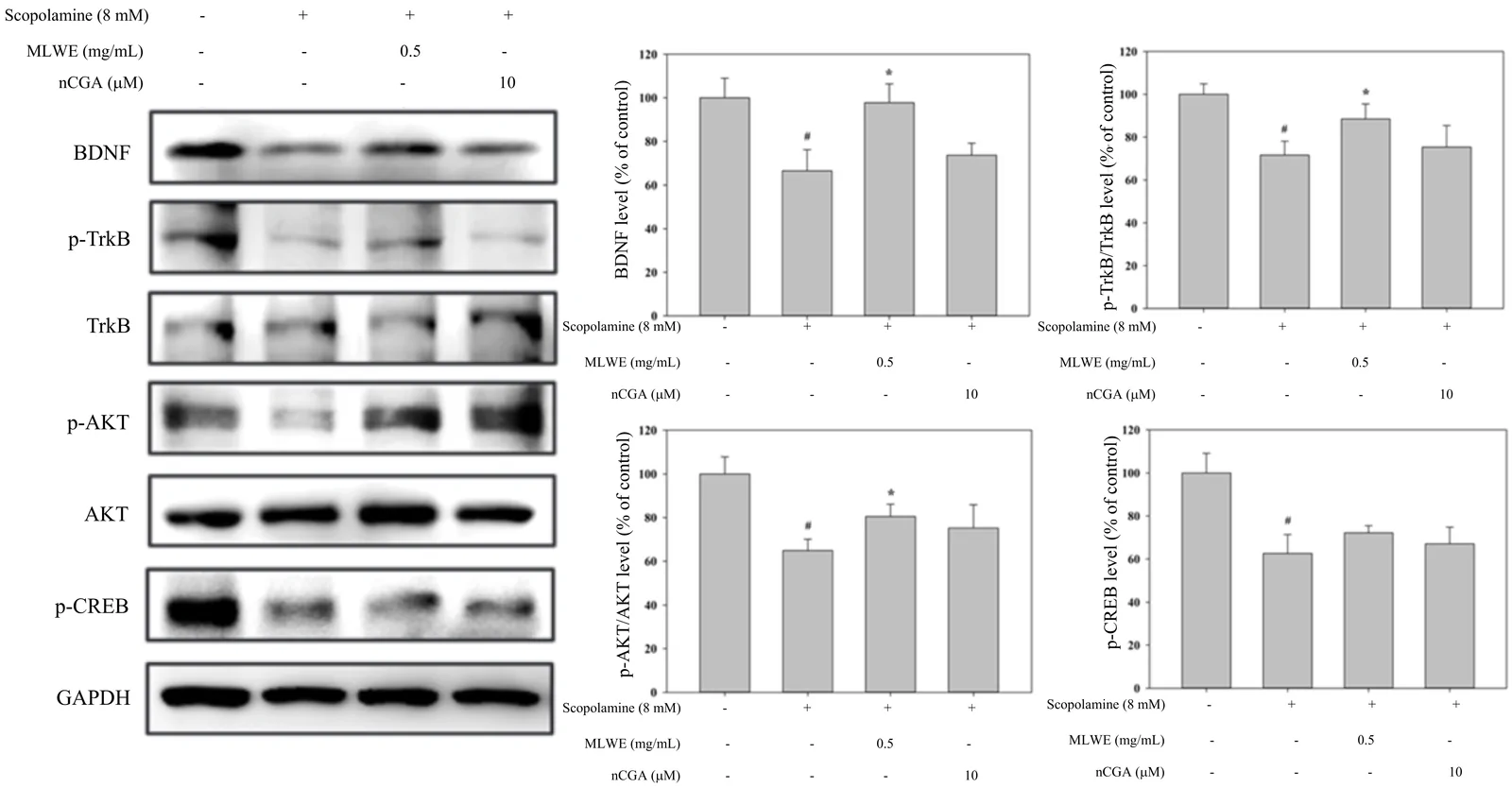
MLWE but not nCGA promoted BDNF pathway of SH-SY5Y cells in response to scopolamine treatment. SH-SY5Y cells were treated with scopolamine combined with MLWE or nCGA for 24 h, the expression of BDNF, phosphorylated Trk B, phosphorylated AKT, and phosphorylated CREB was analyzed by Western blot analysis. Data are shown as means ± SD. # p < 0.05, compared with the control group and * p < 0.05, compared with the Scopolamine group.

**Figure 8:**
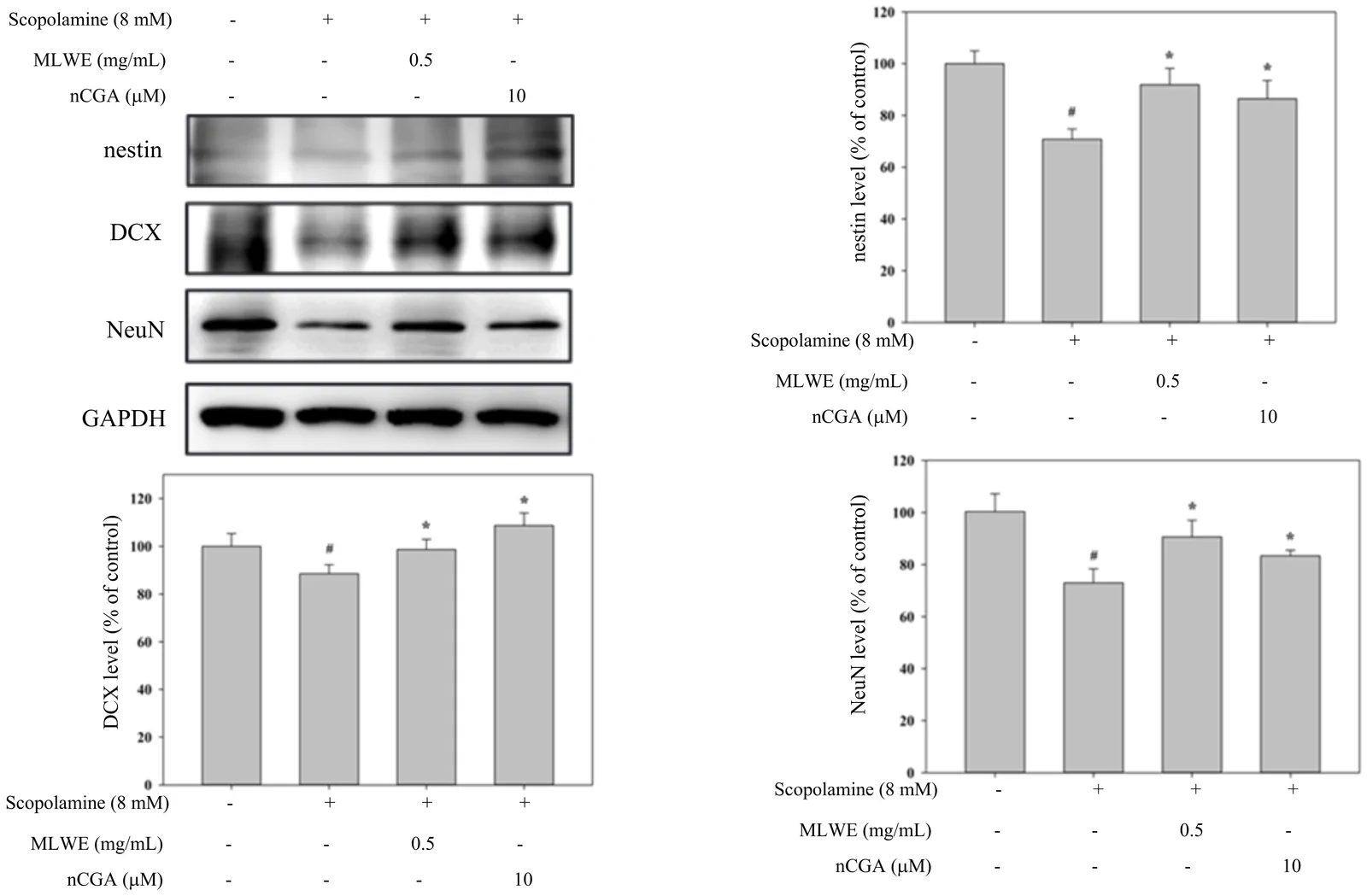
MLWE and nCGA elevated neurogenesis marker expression in scopolamine treated SH-SY5Y cells. SH-SY5Y cells received indicated treatment was subjected into Western blot analysis using anti-nestin, anti-DCX, and anti-Neu N antibodies. Data are shown as means ± SD. # p < 0.05, compared with the control group and * p < 0.05, compared with the scopolamine group.

## Data Availability

All other data are available from the corresponding author upon reasonable request.
