# Supplementary material for: Mulberry Leaf Extracts and Neochlorogenic Acid Ameliorated Scopolamine-induced Cognitive Dysfunction through Promotion of Adult Hippocampal Neurogenesis
Source: Int J Med Sci. 2026 Aug 24;23(10):3156–68. doi: 10.7150/ijms.136787 (PMC13617504; doi:10.7150/ijms.136787)
Supplement: Supplementary file 1 [file ijmsv23p3156s1.pdf]

Supplementary Fig. 1.

(A)

	Control	Scopolamine	Tacrine	MLWE	nCGA
**AST (U/L)**	60.88 ± 8.8	59.88 ± 18.3	68.88 ± 17	63.25 ± 10.41	61.63 ± 10.95
**ALT (U/L)**	34.25 ± 5.04	32 ± 6.69	34.63 ± 5.63	29.38 ± 2.64	33.63 ± 4.27
**BUN (mg/dL)**	28.49 ± 5.47	28.75 ± 5.12	23.65 ± 4.31	20.2 ± 3.59	22.23 ± 3.94
**CRE (mg/dL)**	0.49 ± 0.03	0.46 ± 0.05	0.45 ± 0.1	0.48 ± 0.04	0.45 ± 0.1
**UA (mg/dL)**	4.8 ± 1.04	4.45 ± 0.59	4.24 ± 0.59	3.99 ± 0.78	4.11 ± 0.45
**Cholesterol (mg/dL)**	87.13 ± 6.49	91.38 ± 14.47	85.25 ± 11.49	85.75 ± 11.97	91.13 ± 11
**TG (mg/dL)**	82.38 ± 25.89	80 ± 27.54	65.63 ± 17.45	59.25 ± 18.01	57 ± 11.81
**Glucose (mg/dL)**	347 ± 53.16	328 ± 44.42	312.25 ± 56.18	299.38 ± 64.69	311.88 ± 23.3

(**B**)

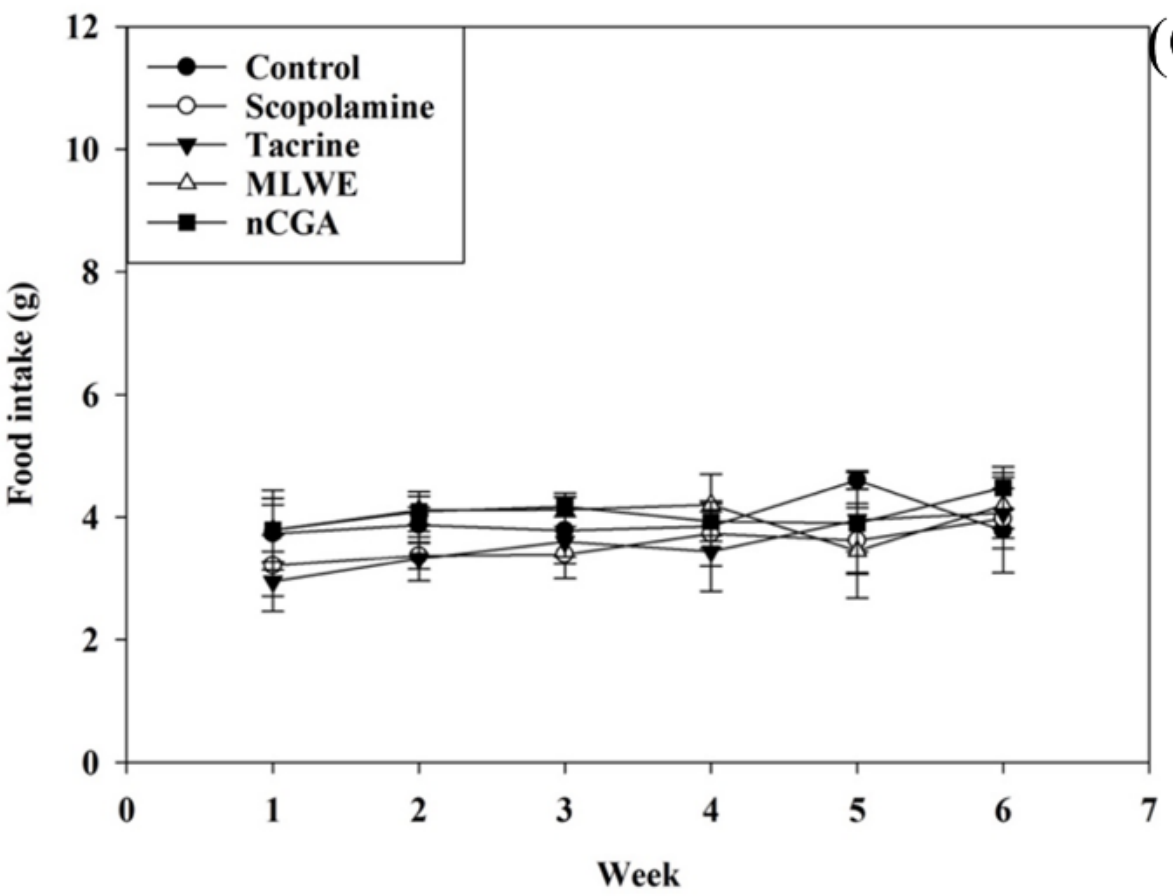

(C)

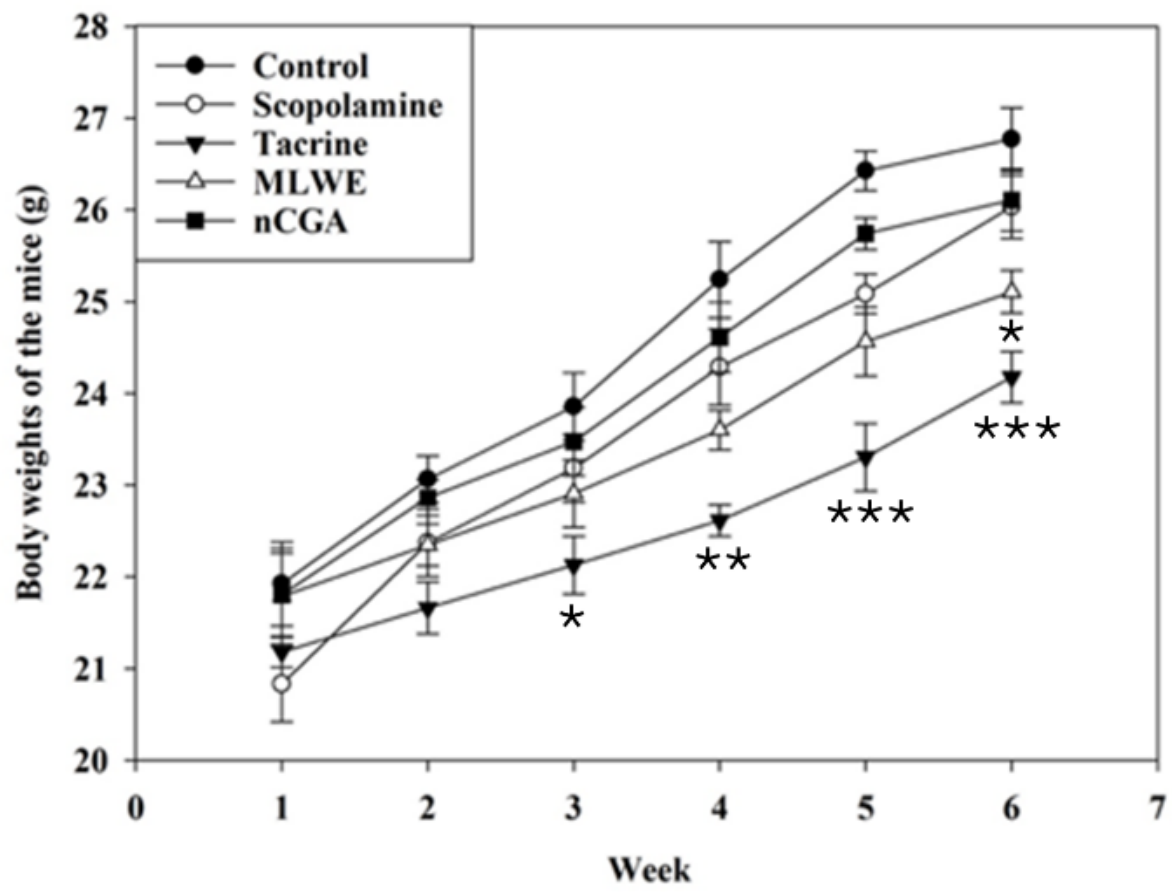

Supplementary figure 1. The effect of MLWE and nCGA on (A) plasma biochemical parameters (B) food intake and (C) the body weight change in scopolamine-induced C57BL/6 mice. C57BL/6 mice were fed with indicated diets as mentioned in material and method section for six weeks. Data represented means ±SD. * $p < 0.05$; ** $p < 0.01$, ***: $p < 0.005$ compared with the control group.

Supplementary Fig. 2

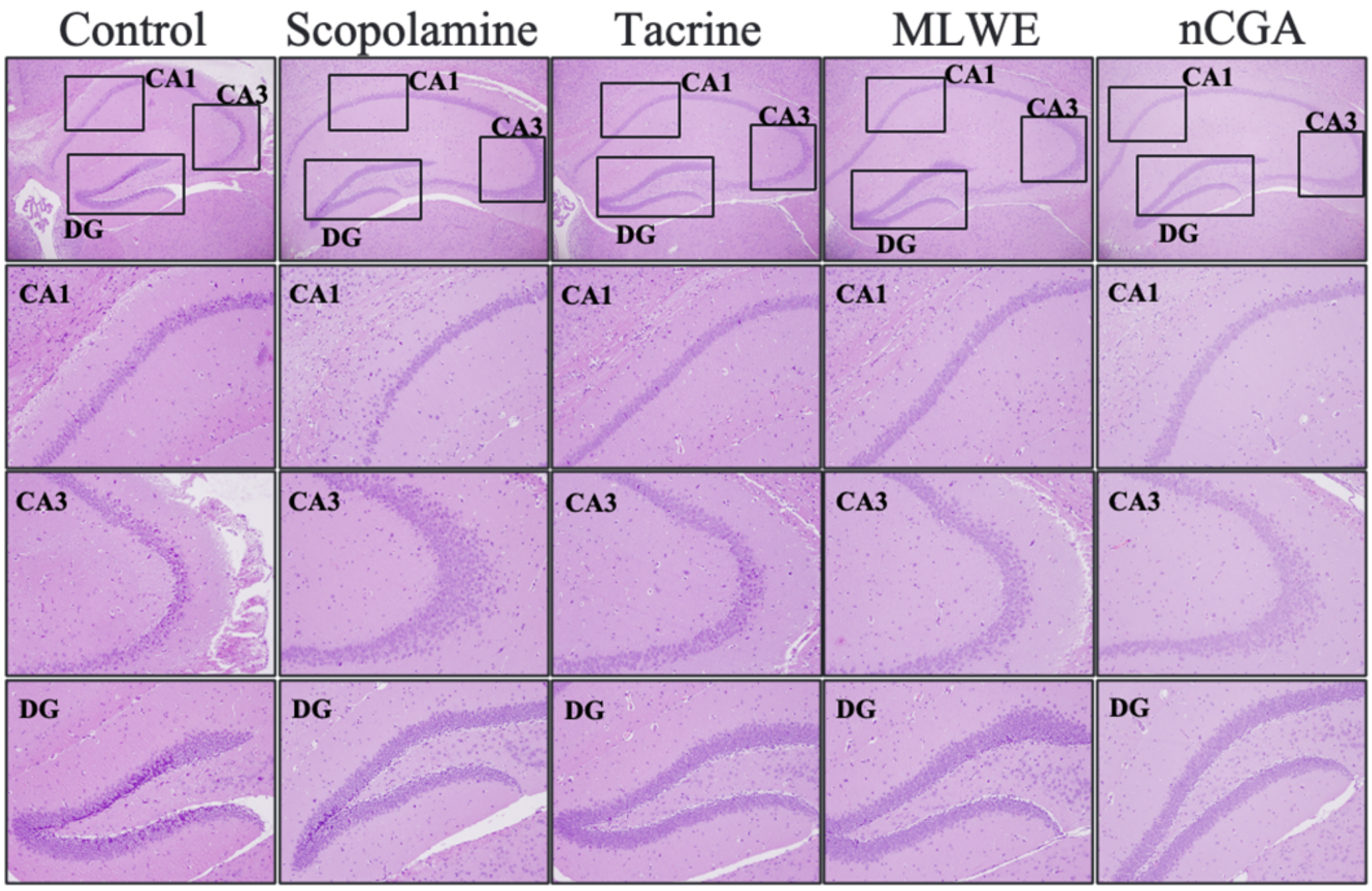

Supplementary figure 2. Representative H&E-stained sections of the hippocampal structure in scopolamine-induced mice. Hippocampus region from mice fed with indicated diet as mentioned in material and method section for six weeks. Brain sections were stained with H&E. The images were captured under a microscope with magnification at 100X, 200X.

Supplementary Fig. 3

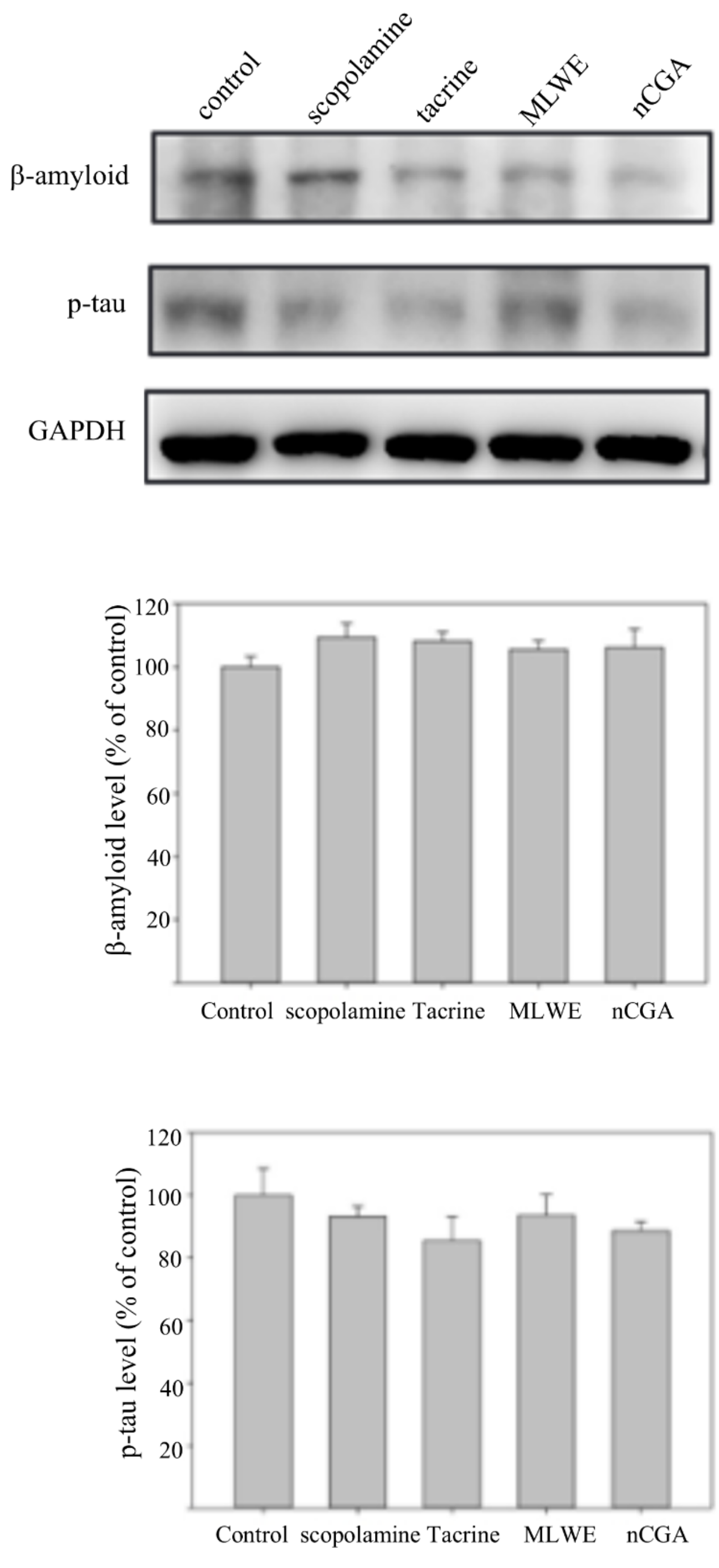

Supplementary Fig. 3. No effects of MLWE and nCGA on β-amyloid and phsphorylated tau (p-tau) expression. Hippocampus tissue extracts from indicated treated groups as mention in materials and methods section were subjected to Western blotting to analyze β-amyloid and phsphorylated tau (p-tau) expression. Data are shown as means ± SD.
